# Heterogeneity of white-matter organization in the human brain

**DOI:** 10.64898/2026.03.31.714863

**Published:** 2026-04-03

**Authors:** Emily Turschak, Wan-Qing Yu, Kevin Takasaki, Steven J Cook, Russel Torres, Olga Gliko, Ayana Hellevik, Kareena Villalobos, Elizabeth Guadarrama, Soumya Chatterjee, Eric Perlman, Connor Laughland, Adam Glaser, Uygar Sümbül, R Clay Reid

## Abstract

White-matter, which contains the long-range axons connecting different brain regions, makes up nearly half the human brain, yet the three-dimensional organization of individual axons has not been characterized. While advances in diffusion MRI have enabled macroscopic mapping of major WM pathways^1^, these methods are unable to resolve individual axons: their trajectories, density, and relative orientations^2^. Here, we present a histological and imaging pipeline optimized for post-mortem human white-matter that shows the 3D trajectories of densely stained large (diameter greater than ~1 μm) projection axons. Applying our approach to multiple centimeter-scale WM regions in an adult human brain, we observed striking regional diversity in axonal organization. Specifically, we identified distinct architectural motifs ranging from loosely packed, multi-orientation meshworks (in most superficial white matter), to laminar lattice-like structures (near the basal ganglia), to tightly packed bundles of fibers (e.g. in the corpus callosum). We speculate that these patterns reflect local adaptations to spatial constraints, axonal density, and the diversity of axonal sources and targets, offering region-specific solutions to anatomical optimization problems. These findings offer new insights into the principles shaping brain connectivity and underscore the need for regionally detailed atlases of human white matter..

White-matter (WM) accounts for nearly half of the human brain’s volume, a proportion that increases with brain size, making up only 12% of mouse and 25% of macaque brains^3^. While studies of neuroanatomy, primarily in experimental animals and to a lesser extent in humans, have yielded remarkable insights into mammalian brain architecture, comprehensive and high-resolution datasets characterizing WM organization in the human brain do not exist. This lack is largely due to persistent technical limitations in both microscopy and histological processing.

In fact, prior to recent advances^4,5^, axons in human WM could be traced over only very short (<1 cm) distances^6^, owing to technical limitations in both microscopy and histological processing. Direct anatomical studies of WM microstructure have long faced a tradeoff between resolution and spatial extent. For example, electron microscopy (EM) provides nanometer-level detail but is limited to small tissue volumes (≤1 mm^3^), whereas magnetic resonance imaging (MRI) can image whole brains but lacks the resolution to resolve individual axons. While large-scale initiatives such as the Human Connectome Project have generated valuable maps of macroscopic WM tracts using diffusion MRI (dMRI) and tractography^1,7^, these methods yield only indirect, model-based estimates of axon trajectories^8,9^.

These technical barriers have long been recognized as a fundamental obstacle to understanding the human brain. Over 30 years ago, Francis Crick and Edward Jones^10^ decried “The backwardness of human neuroanatomy” compared to pathway tracing studies in experimental animals. Their blunt assessment still rings true today: “*The shameful answer is that we do not have such detailed maps because, for obvious reasons, most of the experimental methods used on the macaque brain cannot be used on humans*.” After decades of research, our understanding of the microstructure of human WM remains extremely limited, as we still lack techniques capable of tracing long-range axons at single-fiber resolution or revealing their fine-scale spatial organization within human WM.

Advances in histological processing (including hydrogel-based tissue expansion^11–17^ and optical clearing^18–23^ and in microscopy techniques such as high-throughput lightsheet imaging^24^ have begun to overcome these barriers^4^. However, human WM remains particularly challenging due to its dense architecture and light-scattering properties. Continued development of scalable, single-axon resolution imaging pipelines are essential for building anatomically accurate, regionally detailed maps of human WM organization, an endeavor that stands to significantly advance our understanding of brain connectivity in health and disease^25,26^.

We ultimately aim to construct a projection map of the human brain at single-axon resolution. As a step toward this goal, we demonstrate the diverse local organization of axon trajectories across human WM regions, extending observations from previous histological studies and inferences from dMRI^27–32^. Our results demonstrate that human WM is far from uniform in its microarchitecture, but instead is organized into strikingly distinct regional motifs that reflect local anatomical and functional demands. These findings establish a foundation for understanding lower-resolution approaches, such as dMRI, and for mapping brain connectivity at single-axon resolution.

## Methods

**Figure 1.**
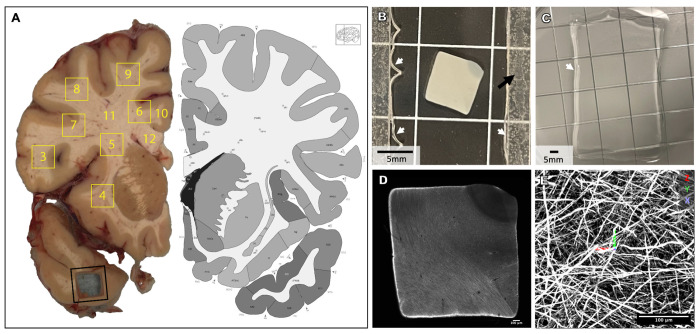
(A) Fixed hemi-coronal brain slab (~0.5 cm thick), with a representative ~0.6 cm^2^ punchout removed from the temporal lobe (black outline) to illustrate the sampling approach. Yellow outlines and labels indicate the locations of the sampled regions. Punchout 2 is not shown. Punchouts 3–9 were obtained using the punch tool, whereas 11 and 12 were dissected from the remaining tissue with scalpel cuts. Corresponding atlas image from Allen Institute website resource. (B) 500 μm section from punchout #6. White arrows = gel border. Black arrow = spacer for polymerization chamber. (C) Cleared tissue-hydrogel matrix, expanded in water. White arrow = gel border. (D) Confocal overview of polymerized section, pre-expansion. (E) ExA-SPIM lightsheet image example. Scale bars relative to actual size, not corrected for expansion.

We developed a pipeline combining antibody labeling, optical clearing, hydrogel expansion, and light-sheet imaging to resolve individual axons across centimeter-scale volumes of post-mortem human WM (see Supplementary Methods). The tissue processing protocol integrated elements from tissue preservation strategies (eg. SHIELD^23^, LICONN^16^), tissue delipidation and optical clearing (eg., LifeCanvas delipidation: Park 2019; CUBIC^22^), and hydrogel-based isotropic expansion (eg. ExM^11^, ELAST^14^, LICONN^16^. Flash-frozen coronal slabs (0.5 cm) of an adult human brain^33^ were fixed and cut into ~0.6x0.6x0.5 cm tissue volumes (“punchouts”) (Fig. 1A), and subsequently sectioned at 500 μm (Fig. 1B) on a Leica VT 1000S vibratome. Sections were incubated in a polyepoxide-based stabilization solution (SHIELD) and delipidated with Clear+ buffer (*LifeCanvas Technologies*^23,34^. Neurofilament heavy protein (NFH) was labeled using our adapted immunolabeling protocol. NFH was selected based on its established use for myelinated long-range projection axons^35–38^. Control experiments with viral labeling of excitatory neurons in macaques demonstrated that all virally labeled cortical axons were also labeled with NF antibodies (data not shown *[can be included in revision])* Samples were then embedded in a tissue-hydrogel matrix and isotropically expanded to 3x their original size (Fig. 1C, D). Expanded gels were adhered to polylysine-coated glass slides and mounted to a sample arm using epoxy-protected magnets, followed by imaging with an ExA-SPIM^24^, a high-speed, large-field-of-view light-sheet microscope.

## Results

We examined NFH labeled axons across samples to identify shared features and characterize regional and structural variability in axonal organization. NFH labeling produced strong signal in axons, with minimal background staining. There was considerable spacing between axons, which enabled most with larger caliber (above ~1.5 μm in diameter) to be readily traced manually with few ambiguities. Here, we address local WM structure rather than long-range connectivity, so detailed analysis of axon segmentation and traceability is deferred to a more technical report^5^.

**Figure 2.**
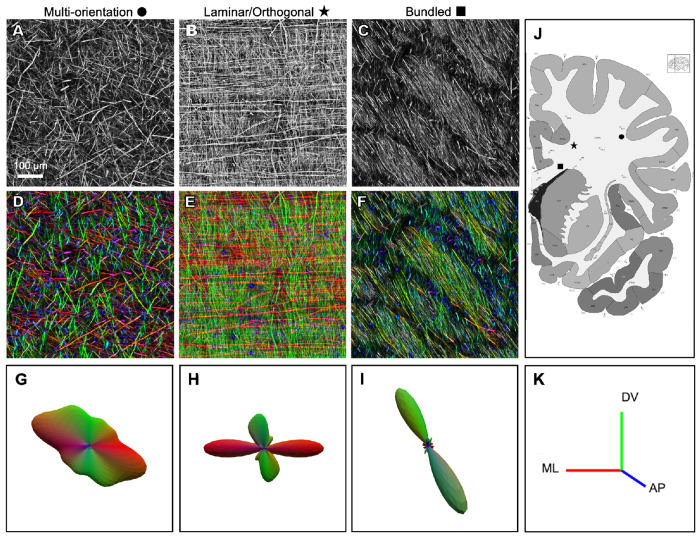
Visualizing the data with structure tensor analysis (A-C) Representative examples of three distinct organizational motifs: (A) multi-orientation, (B) laminar/orthogonal, and (C) bundled, shown in grayscale. (D-F) Corresponding orientation maps derived from structure tensor analysis, with color indicating local axonal orientation (D: multi-orientation, E: laminar/orthogonal, F: bundled). (G-I) ODFs for each example, illustrating the distribution of axonal orientations (G: multi-orientation, H: laminar/orthogonal, I: bundled). (J) Coronal atlas section corresponding approximately to the sampled tissue slab. (K) Structure tensor color key, with red indicating medio-lateral, green dorso-ventral, and blue antero-posterior orientations.

Our findings highlight striking regional diversity in axonal organization across human WM, which we refer to here as multi-orientation, laminar/orthogonal, and bundled (Fig. 2A–C). The statistics of local axonal organization can be quantified with structure tensor analysis, a method widely applied in image processing, computer vision, and biomedical imaging, to characterize fiber orientations^39–41^. Structure tensor analysis enables color-coding of axons based on their local orientation, with red coding for medio-lateral, green for dorso-ventral, and blue for antero-posterior orientations (Fig. 2D–F). The distribution of orientations at a location yields a 3-dimensional orientation distribution function (ODF; Fig. 2G–I). In the case of the multi-orientation domains (Fig. 2A, D) the ODF typically has multiple broad peaks (Fig. 2G), while the laminar/orthogonal domains (Fig. 2B, E) has two peaks (Fig. 2H).

The multi-orientation organization was found in superficial WM, near the cortical surface, characterized by relatively low density of NFH-labeled axons (Fig. 2A), multiple orientations (Fig. 2D), and an absence of discernible local bundling (Fig. 2J), resulting in a seemingly random three-dimensional meshwork. Further from the cortical surface, such as the broad expanse of WM known as the centrum semiovale, the multi-orientation mesh-like structure was observed, although with mixtures of orientations that varied gradually across the region (see below).

In other WM regions, we observed several varieties of more structured axonal organization. The laminar/orthogonal organization was seen at the border of the centrum semiovale, near the basal ganglia and corpus callosum. Most axons had one of two orthogonal orientations^42^, with a laminar organization (which predominates in mouse WM^43^) that appeared almost woven. (Fig. 2B, J, and see below). This may represent an alternate organizational solution for routing axons through constrained spaces, possibly balancing structural coherence with the need to accommodate multiple trajectory angles within the same volume.

**Figure 3.**
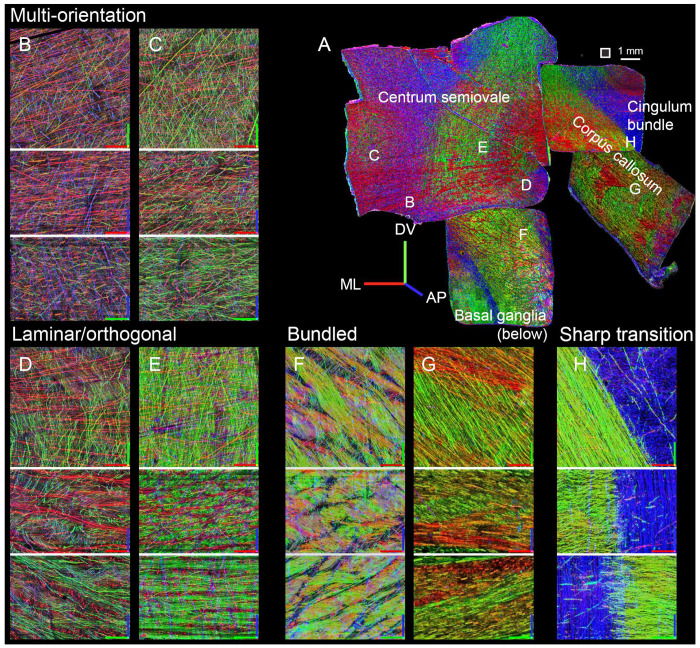
Examples of each type of WM organization. (A) Montage of the four central punchouts(11, 6, 12, and 5), manually arranged to approximate their spatial positions within the original tissue slab.(Fig. 1A). Square next to scale bar indicates the size of regions (500 μm) in the remaining panels . (B-H) Three orthogonal views through the middle of 500x500x350 μm image stack, shown as maximum intensities, projected through 60 μm (B-E) or 30 μm (F-H; because of the higher density of bundled axons). Axes are color-coded as follows: red=medio-lateral, green=dorso-ventral, blue=antero-posterior. The red-green plane represents the top-down view of the samples. Axis length = 100. Panels are grouped by configuration. (B-C): Multi-orientation, with individual, ungrouped axons coursing in different directions. ; (D, E): Laminar/orthogonal, with lamination visible in the bottom two projections, in the AP/blue direction. (F, G): Bundled, above the basal ganglia and in corpus callosum, respectively. (H) Border between bundled organization in the corpus callosum and multi-orientation (but with a predominance of the antero-posterior orientation) in cingulum bundle.

The bundled organization was seen in several locations. Near the basal ganglia, (Fig. 2C, F) we observed intertwined bundles, each with largely parallel axons, but with slightly different orientations and different apparent densities. One hypothesis is that the sparser bundles contain a mixture of axons with differential antibody staining. The bundled organization was also seen in the corpus callosum, where all axons had similar orientations, but organized in smaller bundles of nearly parallel fibers (see Fig. 3G, below). This organization is consistent with prior descriptions of the corpus callosum as a high-throughput commissural pathway, composed primarily of long-range axons that connect homologous cortical areas across hemispheres^27,32,44–48^.

To further illustrate these different modes of organization, we offer examples from the four most central punchouts, including the corpus callosum, its border with the cingulum bundle, the centrum semiovale, and its inferior bordering region near the basal ganglia (punchouts 12, 6, 11, and 5, respectively). The multi-orientation meshwork was the predominant pattern observed in portions of the centrum semiovale near the cortical surface (Fig. 3B, C), while the laminar/orthogonal pattern was seen in adjacent positions nearer the corpus callosum (Fig. 3D, E). As noted above, bundled organization was seen near the basal ganglia, just superior to the external capsule (Fig. 3F) and within the corpus callosum (Fig. 3G*)*. Axon density increased progressively from multi-orientation to laminar/orthogonal to bundled configurations. At the interface between the corpus callosum and adjacent WM near the cingulum bundle, we observed an abrupt transition between the tightly bundled axonal architecture of the corpus callosum and the cingulum bundle (Fig. 3H), which despite the high density of antero-posterior axons, had isolated axons coursing with other orientations.

**Figure 4:**
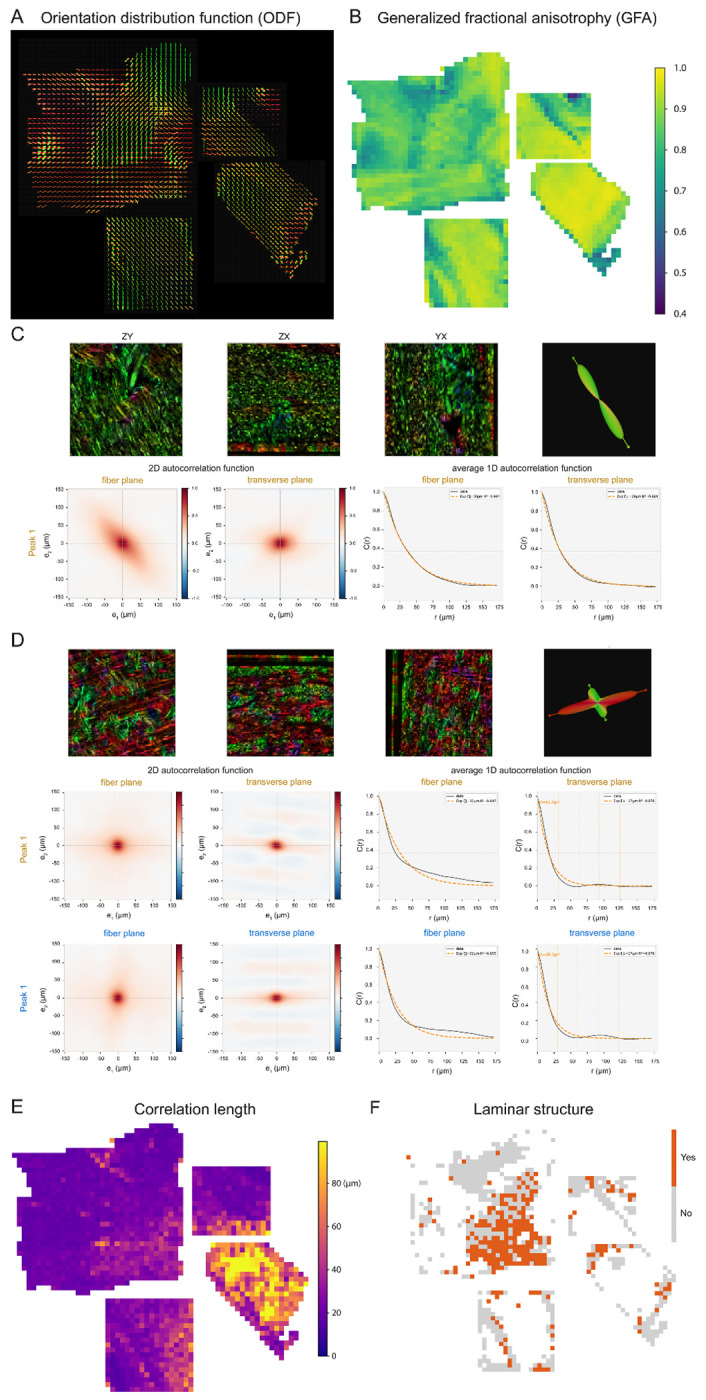
Multiscale quantitative characterization of fiber organization across human WM regions. (A) 3D orientation distribution functions (ODF) glyphs per 0.5 mm^3^ block. Glyph shape reflects the degree of directional coherence, with elongated forms indicating aligned fiber populations and multi-lobed forms indicating crossing or dispersed fiber arrangements. (B) Spatial heatmap of generalized fractional anisotropy (GFA; range 0.4-1.0) quantifying regional variations in orientation coherence. Higher values (yellow) indicate strongly aligned fiber tracts; lower values (purple) reflect isotropic or complex fiber architecture. (C, D) Characterization of local spatial arrangements of fibers via autocorrelation function analysis. (C) Representative coherent tract motif with tightly aligned fibers, yielding a single dominant ODF peak and smooth exponential decay in both 2D and radially averaged 1D autocorrelations in the fiber and transverse planes. (D) A representative crossing orthogonal fiber with laminar structure, yielding a two-peak ODF. Notably, both the 2D and average 1D autocorrelation in the transverse plane exhibit spatial oscillations, providing quantitative evidence of a periodic, laminar-like fiber arrangement rather than random intermingling. (E) Spatial heatmap of maximum autocorrelation length, revealing the physical scale over which the fiber orientation remains coherent. Regions with long correlation lengths (yellow) correspond to large, organized fiber bundles; short correlation lengths (purple) reflect rapid change of spatial organization. (F) Binary map showing the multi-peak regions containing laminar structure (orange = present, grey = absent)

To quantify these observations, we analyzed the same four most central punchouts (Fig. 4A) by first calculating the structure tensor orientation distributions in 0.4 x 0.4 mm regions (Fig. 4A). We quantified these distributions using generalized fractional anisotropy (GFA), a metric commonly used in dMRI that reflects the degree of axonal alignment, ranging from 0 (all orientations equally represented) to 1 (a single dominant orientation).

Mapping this metric across these punchouts (Fig. 4B) revealed distinct spatial domains, distinguishing the highly parallel arrangement (high GFA) in the corpus callosum from regions of complex, multidirectional dispersion (low GFA). To quantitatively determine the spatial organization of fibers across regions, we applied autocorrelation functions on vector fields derived from structure tensor analysis (Fig. 4C–D). In bundled regions characterized by a single dominant ODF peak, such as the corpus callosum, the autocorrelation exhibited a monotonic decay without periodicity (Fig. 4C). Conversely, in laminar/orthogonal regions containing alternating layers of orthogonally oriented fibers, the 2D and average 1D autocorrelation in the transverse plane revealed distinct spatial oscillations (Fig. 4D). Extending this analysis across the four punchouts, we generated spatial heatmaps of the maximum autocorrelation length. This metric mathematically defines the spatial scale of organization, ranging from approximately 20 μm to over 90 μm. Finally, to delineate the spatial extent of these laminar domains, we generated a binary map based on the presence or absence of periodic autocorrelation oscillations, requiring detection of at least two orientation peaks in the ODF. This analysis clearly revealed that the region of the centrum semiovale nearest the callosum and the basal ganglia (lower right) comprises widespread, contiguous domains of structured, laminar-like fibers across several millimeters of tissue.

## Discussion

We applied our pipeline to centimeter-scale WM regions from an adult human brain. Our findings highlight striking regional diversity in axonal organization across the WM, including a multi-orientation meshwork in superficial WM, near the cerebral cortex (Fig. 2A), orthogonally oriented trajectories arranged in a laminar organization trajectories in regions near the basal ganglia (Fig. 2B), and tightly bundled, coherent fiber arrangements in and near the corpus callosum (Fig. 2C).

Histological studies have long suggested that WM microstructure varies across brain regions^27,28,43,49,50^, reflecting underlying circuit specialization. For example, differences in axon diameter, packing density, and myelin sheath thickness are well-documented in the human corpus callosum ^27,32,45,47^, which is well known for having densely bundled axons with a limited range of orientations^44,48^. Similar diversity in axon caliber has also been observed across some cortical and commissural pathways^46^, aligning with the notion that WM architecture is shaped by the distinct functional demands of each tract.

While the biological basis for these organizational motifs remains incompletely understood, several hypotheses grounded in anatomical and geometric principles may help explain this variation. One likely factor is the spatial density and physical constraints of the local WM environment. In regions of high axonal density, bundled, parallel fiber arrangements may offer an efficient solution to packing large numbers of axons into compact volumes. Prior theoretical work suggests that such bundling may reduce wiring length and metabolic cost while maintaining large diameters required for high conduction velocity^28,51,52^. This is consistent with observations in the corpus callosum and internal capsule, where tightly aligned fibers likely reflect a drive toward wiring optimization under strong spatial constraints.

In other regions (such as in portions of the centrum semiovale, Figs. 2A, 3C, 3D), we observed a laminar organization: stacks of layers with alternating, nearly orthogonal fiber orientations. Rather than forming parallel fascicles, which by definition are not planar structures, the layers maintained relatively consistent spacing and orientations (as quantified in Fig. 4), although the layers frequently intermingled (Fig. 3C, D), producing woven or lattice-like configurations. These layers present an alternate organizational solution for routing axons through moderately dense regions, possibly balancing structural coherence with the need to accommodate multiple angles within the same volume. The predominance of near-orthogonal trajectories was noted previously^30,42^, but the laminar organization appears novel.

Finally, in areas with lower axonal density (such as subcortical WM) axons may be less tightly constrained to achieve dense packing. This can give rise to more heterogeneous, multi-orientation configurations, where axons traverse the tissue along independently varying paths. This uncorrelated meshwork organization may present an optimal solution for accommodating multiple pathways that overlap en route to diverse cortical or subcortical targets^46^. Such arrangements resemble the intersection zones described in dMRI studies as sites of high orientation dispersion^53^. Theoretical models have proposed that axonal trajectories reflect a balance between minimizing total wiring length and preserving topographic specificity, which has been termed the “wiring economy” principle^45,54–56^. From this perspective, the observed regional differences in organization may emerge from local solutions to a high-dimensional spatial optimization problem, constrained by both physical space and the complexity of circuit topology.

Although further work is needed to formalize and test these hypotheses, our findings demonstrate that human WM is highly variable in its organization, exhibiting region-specific microarchitectural adaptations that may reflect differences in functional demands, developmental trajectories, and spatial constraints. These patterns will have important implications in multiple areas: (1) for understanding how large-scale brain networks are physically constructed, (2) for interpreting disruptions to WM organization in neurodevelopmental and neurodegenerative disease^25,26^, (3) to inform the calibration and interpretation of lower-resolution techniques, such as dMRI and optical coherence tomography, and (4) to guide neurosurgical approaches that depend on accurate delineation of white-matter architecture.

## Supplementary Material

1
