# Supplementary material for: Heterogeneity of white-matter organization in the human brain
Source: bioRxiv. 2026 Apr 3:2026.03.31.714863. Preprint. [Version 1] doi: 10.64898/2026.03.31.714863 (PMC13060348; doi:10.64898/2026.03.31.714863)
Supplement: 1 [file NIHPP2026.03.31.714863v1-supplement-1.pdf]

bioRxiv preprint doi: https://doi.org/10.64898/2026.03.31.714863; this version posted April 3, 2026. The copyright holder for this preprint (which was not certified by peer review) is the author/funder, who has granted bioRxiv a license to display the preprint in perpetuity. It is made available under aCC-BY 4.0 International license.

SUPPLEMENTARY METHODS

Histology

Tissue Source, Slab Preparation, and Sectioning

Postmortem human tissue from an adult male donor (61 years old, Hispanic, with no known neuropsychiatric or neurological conditions) was obtained from the San Diego Medical Examiner's Office. The brain was processed by bisecting at the midline, as described in[33], and coronal slabs approximately 0.5 cm thick were embedded in alginate, rapidly frozen in a dry ice-isopentane slurry, vacuum sealed, and stored at -80°C. When retrieved for processing, the frozen slab was drop-fixed in ice-cold 4% paraformaldehyde (PFA) and 10% acrylamide in phosphate-buffered saline (PBS) for 24 hours at 4°C with gentle agitation (as adapted from the LICONN protocol). The fixed tissue was then rinsed overnight in 100mM glycine in 1×PBS at 4°C to quench residual aldehydes[34], followed by 3 rinses (>15min each) in 1×PBS at 4 °C with gentle agitation.

Individual 1cm³ regions of interest (Fig. 1A) were sampled from the slab using a square punch tool. Each tissue block was embedded in 4% agarose in 1X PBS and sectioned at 500 µm on a Leica VT 1000S vibratome for downstream processing. Individual sections were transferred to a 15 mL tube, where all subsequent processing steps were carried out to minimize tissue handling and reduce contamination risk. Sections remained in their respective tubes until polymerization.

Post-Fixation

(adapted from Park et al., 2018)

Tissue sections were incubated in a polyepoxide-based stabilization method adapted from the SHIELD protocol[23], which preserves protein antigenicity and tissue architecture during downstream clearing and expansion. Sections were incubated in SHIELD-Off buffer for 72 hours at 4°C with gentle agitation, followed by incubation in SHIELD-On (*LifeCanvas Technologies*) buffer for 24 hours at 37°C with gentle agitation. This step minimizes tissue degradation and molecular loss during subsequent processing, enabling consistent preservation across large volumes of human WM. After SHIELD stabilization, sections were rinsed in 1xPBS three times for 15 minutes each.

Delipidation

Tissue sections were delipidated using Clear+ buffer (*LifeCanvas Technologies*[23,34]) for 2 weeks at 37 °C with gentle agitation. Buffer volume was supplemented after one week to ensure adequate penetration (3mL initial +

bioRxiv preprint doi: https://doi.org/10.64898/2026.03.31.714863; this version posted April 3, 2026. The copyright holder for this preprint (which was not certified by peer review) is the author/funder, who has granted bioRxiv a license to display the preprint in perpetuity. It is made available under aCC-BY 4.0 International license.

3mL additional). Following delipidation, samples were rinsed in 1×PBS containing 0.02% sodium azide to remove residual clearing agents. Sodium azide was included in this rinse step to prevent microbial growth.

Antibody labeling

Blocking was performed in NGSTU-azide buffer (prepared by diluting 10×PBS to 1× with NGSTU-azide buffer) for 24 hours at room temperature. Urea was included in the blocking and primary antibody buffer solutions to enhance tissue permeability, an approach inspired by CUBIC protocols[33]. Primary antibody incubation was carried out using anti-NFH antibody (see below) (1:500) in NGSTU-azide buffer. Samples were incubated sequentially under the following conditions: 2mL for 1 week at 4°C, an additional 2mL for 1 week at room temperature, and a final addition of 2mL for 1 week at 37°C, all with gentle shaking. Following primary antibody incubation, tissue was rinsed with 5mL of 1×PBS-azide a minimum of four times for 15 minutes each. For the final rinse, tissue was incubated in 10mL of 1×PBS-azide overnight at 4°C. Secondary antibody incubation used 1:250 goat anti-mouse Alexa Fluor 488 antibody, see below*)* in NGST-azide buffer. Urea was not included in the secondary antibody buffer solution due to concerns of fluorescence quenching. Sections were incubated with 4mL of secondary antibody solution for 1 week at room temperature, with an additional 2 mL added at the one-week mark, followed by incubation for 1 more week at 37°C (final volume: 6 mL). Following secondary antibody incubation, tissue was rinsed with 5 mL of 1×PBS a minimum of five times for 15 minutes each, then transferred to 10 mL of 1×PBS overnight at 4°C. Sodium azide was removed from this rinse step to prevent interference with downstream hydrogel polymerization (it is highly reactive, which could potentially interfere with or destabilize the desired reaction products). All incubations steps, including rinses, were done with gentle shaking.

Chemical Anchoring

(adapted from Tillberg et al., 2016)

Acryloyl-X, SE (AcX) was used to covalently anchor amine-containing biomolecules to link them to the expanding polymer matrix (see below). AcX was first resuspended in anhydrous DMSO to a concentration of 10 mg/mL, aliquoted, and stored at −20 °C in a desiccated environment for up to 2 months.

For anchoring, tissue sections were incubated in 0.1 mg/mL AcX diluted in 1xPBS, pH6, for at least 72 hours at 4°C with gentle agitation. AcX treatment introduces acrylamide moieties to endogenous proteins, enabling subsequent covalent linkage to the polymer network during hydrogel polymerization. Tissue was then rinsed in 1xPBS five times for 15 minutes each.

Expanding Hydrogel Monomer Incubation

Prior to gelation, the expanding hydrogel monomer stock solution[16] was thawed on ice. For each 1 mL of monomer solution, 15 µL of VA-044[14], a thermal initiator, was added.

The solution was vortexed to ensure homogeneity, and 1 mL was added to each tissue section (1 cm × 1 cm × 0.5 mm) in their respective 15 mL conical tubes, ensuring full coverage of the tissue. Samples were incubated for at least 72 hours at 4 °C with gentle agitation to allow thorough diffusion of the monomer into the tissue.

Immediately prior to polymerization, tube caps were removed and samples were placed in a vacuum desiccation chamber for 15 minutes to degas the solution and minimize oxygen inhibition of polymerization.

Chamber construction and polymerization

Polymerization chambers were constructed by placing a 0.5mm adhesive spacer (16x26mm inner; 22x30mm outer; see Fig. 1B) on a microscope slide. The chamber was filled with monomer solution and tissue was placed in the center of the spacer. A 24x55mm #1 coverslip was then carefully placed on top

bioRxiv preprint doi: https://doi.org/10.64898/2026.03.31.714863; this version posted April 3, 2026. The copyright holder for this preprint (which was not certified by peer review) is the author/funder, who has granted bioRxiv a license to display the preprint in perpetuity. It is made available under aCC-BY 4.0 International license.

of the spacer, ensuring that no bubbles were trapped in the solution. Chambers were placed in a 10cm petri dish, which was subsequently gently purged with nitrogen gas for 30 seconds. The perimeter of the petri dishes was parafilmed, and dishes were placed in a 37°C incubator for two hours without agitation.

Once polymerized, each tissue section was imaged on a confocal microscope while still in the chamber to obtain an overview image prior to removal (see Fig. 1D). This post-polymerization, pre-expansion overview image served as a reference for precise size measurements before enzymatic digestion and tissue expansion, enabling accurate calculation of the final expansion factor.

Enzymatic digestion

Tissue-hydrogel matrices were removed from polymerization chambers and transferred to 60x15mm dishes with 10mL 1:100 Proteinase K in the digestion buffer. Samples were then incubated at 37°C overnight with gentle shaking.

Expansion

After enzymatic digestion, gels were carefully transferred to 150X150x25mm dishes and rinsed in 1xPBS three times for 15 minutes each. Gels were transferred into excess volumes of Milli-Q water and incubated for 15 minutes to 2 hours to initiate expansion. This water exchange step was repeated 3-5 times using fresh Milli-Q water until the sample reached ~4x its original size. Gels were then equilibrated in 0.5x PBS, resulting in a 3x final expansion for lightsheet imaging.

Microscopy

Sample mounting and large-format lightsheet microscopy

A custom expansion-assisted selective plane illumination (ExA-SPIM) lightsheet microscope was constructed according to the design and resources described in the original publication [24] and modified for imaging of 500 µm-thick, 3x expanded sections immersed in 0.5x PBS. Expanded gels were adhered to glass slides coated with polylysine which were then mounted with epoxy-protected magnets to a sample arm tilted at 60 degrees and actuated along the tilted axis with an LS-50 motorized linear stage (Applied Scientific Instrumentation). The tilted stage enabled constant velocity scanning through the lightsheet for tile acquisition and the three-axis motorized stage system (MS-8000 and FTP-100, Applied Scientific Instrumentation) effected gross repositioning of the section between tiles. Microscope hardware was controlled by a custom lightsheet device control and image acquisition software package (Voxel, Allen Institute for Neural Dynamics) and image data was compressed and written directly to the next-generation file format zarr version 3 by an open-source data writer (acquire-zarr, Chan-Zuckerberg Institute). Zarr datasets were concurrently uploaded during acquisition to an on-premises, enterprise all-flash data platform (VAST) with acquisition metadata for downstream processing with the HPC cluster.

Individual physical voxels were ~0.75 µm, nearly isotropic, which corresponds to ~0.25 µm relative to the unexpanded tissue, given the 3x expansion.

Computation

Image data preprocessing: deskewing and downsampling for multiresolution pyramid

Raw data volumes generated by the virtual-V ExASPIM acquisition were skewed with respect to the tissue coordinates and raw images are composed entirely of full resolution voxels. Data preprocessing on the HPC cluster resolves these shortcomings by generating output zarr datasets that have been deskewed into the usual XYZ tissue coordinates. The zarr files contained a downsampled image pyramid (MIP levels) which

bioRxiv preprint doi: https://doi.org/10.64898/2026.03.31.714863; this version posted April 3, 2026. The copyright holder for this preprint (which was not certified by peer review) is the author/funder, who has granted bioRxiv a license to display the preprint in perpetuity. It is made available under aCC-BY 4.0 International license.

facilitates visualizing and processing large volumes of data. MIP0 voxels are ~0.25 μm, relative to the original tissue, MIP1 voxels are ~0.50 μm, etc.

Section preprocessing: stitching

Deskewed tiles were first assembled into a volume in the unified coordinate space of the section by applying translation offsets according to the predefined position of the stage in the acquisition. These offsets were further refined by registering image data in the overlapping regions of the tiles. A custom stitching algorithm uses a combination of SIFT and template matching to define informative points from highly downsampled data around which sub-voxel phase correlation is applied to the highest resolution to extract empirical spatial offsets beyond those defined by the coordinates of the microscope stage[5]. These offsets were recorded as metadata allowing the deskewed tiles to be viewed and manipulated as a single, “stitched volume” by standard big data tools, such as Neuroglancer (https://github.com/google/neuroglancer), as well as the downstream processing pipeline.

MIP4: fusion/transformation to get anatomical orientation

For fusion of adjacent tiles, we designed a module suited for the translation of multiple adjacent tiles. Each tile is placed into a common coordinate space using precomputed translation offsets, whether stage or registration derived, which specify the position of each tile's origin within the global volume, and written into the appropriate location in a single output volume. The output is a single consolidated volume ready for downstream processing steps.

There were several punchout volumes adjacent to one another, but processed without regard to their original orientation. The impact of this on the structure tensor analysis is a mismatch of orientation values for axon traces between adjacent volumes. To resolve this issue, we rotated those volumes using a rotation degree derived from visual inspection in Neuroglancer.

Data analysis

For quantitative analysis of fiber architecture, MIP4 image datasets were divided into 100x100x100 voxel cubes. We utilized structure tensor analysis[57] to extract primary fiber orientations from local image intensity gradients, which were then projected onto a 6,500-point spherical space. This generated discrete orientation distribution functions for each cube, allowing us to calculate generalized fractional anisotropy and identify orientation peaks separated at least by 45°. To evaluate the spatial arrangement of these fiber tracts, we calculated the 3D autocorrelation function from the local orientation tensor field. The spatial scale of structural coherence, correlation length, was derived by fitting axial autocorrelation profiles to an exponential decay model. Finally, to systematically map regions of laminar structure of cross fibers without relying on visual inspection, we screened transverse ACF profiles for spatial periodicity. This classification required strict spectral evidence, including ≥1.5 full cycles in the spatial domain coupled with robust signal-to-noise thresholds across both 1D and 2D fourier domains. Tissue cubes with low fractional anisotropy and at the border of the tissues were omitted from all spatial maps.

Neuroglancer links:

Raw

PO12:

https://neuroglancer-demo.appspot.com/#!https://apex-connects.s3.us-east-2.amazonaws.com/axonal_connectomics/contrast_adjusted/H17_PO12_S4_20250501_V2/state2.json

PO6:

https://neuroglancer-demo.appspot.com/#!https://apex-connects.s3.us-east-2.amazonaws.com/axonal_connectomics/contrast_adjusted/H17_PO6_S3_20250422/state2.json

bioRxiv preprint doi: https://doi.org/10.64898/2026.03.31.714863; this version posted April 3, 2026. The copyright holder for this preprint (which was not certified by peer review) is the author/funder, who has granted bioRxiv a license to display the preprint in perpetuity. It is made available under aCC-BY 4.0 International license.

PO5:

https://neuroglancer-demo.appspot.com/#!https://apex-connects.s3.us-east-2.amazonaws.com/axonal_connectomics/contrast_adjusted/H17_PO5_S8_20250410/state2.json

PO11:

https://neuroglancer-demo.appspot.com/#!https://apex-connects.s3.us-east-2.amazonaws.com/axonal_connectomics/contrast_adjusted/H17_PO11_S8_20250408/state.json

Structure tensor MIP4

PO12:

https://neuroglancer-demo.appspot.com/#!https://apex-connects.s3.us-east-2.amazonaws.com/axonal_connectomics/contrast_adjusted/H17_PO12_S4_20250501/equalized_fused/state.json

PO6:

https://neuroglancer-demo.appspot.com/#!https://apex-connects.s3.us-east-2.amazonaws.com/axonal_connectomics/contrast_adjusted/H17_PO6_S3_20250422/equalized_fused/state.json

PO5:

https://neuroglancer-demo.appspot.com/#!https://apex-connects.s3.us-east-2.amazonaws.com/axonal_connectomics/contrast_adjusted/H17_PO5_S8_20250410/equalized_fused/state.json

PO11:

https://neuroglancer-demo.appspot.com/#!https://apex-connects.s3.us-east-2.amazonaws.com/axonal_connectomics/contrast_adjusted/H17_PO11_S8_20250408/equalized_fused_st/state2.json

Fused (lower res)

https://neuroglancer-demo.appspot.com/#!https://apex-connects.s3.us-east-2.amazonaws.com/axonal_connectomics/contrast_adjusted/smlori11-Big-mv960-160_rot100/equalized_fused/state.json

bioRxiv preprint doi: https://doi.org/10.64898/2026.03.31.714863; this version posted April 3, 2026. The copyright holder for this preprint (which was not certified by peer review) is the author/funder, who has granted bioRxiv a license to display the preprint in perpetuity. It is made available under aCC-BY 4.0 International license.

Supplemental: Materials and equipment

Reagents

- Paraformaldehyde 32% aqueous solution EM grade (Electron Microscopy Sciences, #15714-5)
- Glycine (Sigma-Aldrich, #G7126)
- PBS 10x buffer pH7.4 1L (Life Technologies, #AM9625)
- SHIELD kit (SHIELD Epoxy, Buffer, and ON solutions), (LifeCanvas Technologies, SH-250)
- Clear+ delipidation buffer (LifeCanvas Technologies, #DB)
- Agarose (VWR, #IB70042)
- Normal Goat Serum (NGS), (Vector Labs # S-1000)
- Triton X-100 (Sigma-Aldrich, # X100)
- Urea (Sigma-Aldrich, #U2709)
- Sodium Azide, 5% (Fisher Scientific, #71448-16)
- Rabbit anti-neurofilament heavy chain (NF200 or NFH) antibody (Sigma-Aldrich, N4142)
- Goat Anti-Rabbit IgG H&L (Alexa Fluor® 488), (Abcam, ab15007)
- DMSO, Anhydrous (ThermoFisher Scientific, D12345)
- Acryloyl-X (Acx), (ThermoFisher Scientific, A20770)
- Acrylamide (Sigma-Aldrich, #A9099)
- Sodium acrylate, (AK Scientific, #7446-81-3)
- N,N′-(1,2-Dihydroxyethylene) bisacrylamide (Sigma-Aldrich, #294381)
- VA-044 (Fisher Scientific, #NC0632395)
- Sodium dodecyl sulfate (SDS), (Sigma-Aldrich, #L4509)
- 1M Tris-HCl, pH 8.0 (ThermoFisher Scientific, #15568025)
- Proteinase K (ThermoFisher Scientific, #EO0491)
- Poly-L-Lysine (Sigma-Aldrich, #P4707)

Materials/equipment

- ~1 x 1 cm hollow square punch tool (Yizzvb, https://www.amazon.com/dp/B08T94ZLXM)
- Vibratome (Leica VT 1000S)
- Corning® 15ml conical tubes (Millipore Sigma, #CLS430055)
- 50 mL conical tubes
- Epredia™ Richard-Allan Scientific™ Cover Glass, #1, 24x55 (Fisher Scientific,124405)
- iSpacer 0.5mm, (SUNJin Lab Co., #IS002)
- Corning® microscope slides (Millipore Sigma, #CLS294875X25)
- 150X25mm dishes (Fisher Scientific, 08-772-6)
- 100mmx15mm (Fisher Scientific, #263991)
- 60x15mm dishes (Carolina, #741246)
- Olympus FLUOVIEW FV3000
- Lightsheet (ExASPIM)

Solutions

Blocking Buffer and Primary Antibody Buffer

The blocking and primary antibody buffer (NGSTU-azide) was prepared in 1×PBS with the following composition:

- 5% (v/v) normal goat serum
- 0.6% (v/v) Triton X-100
- 4M urea

bioRxiv preprint doi: https://doi.org/10.64898/2026.03.31.714863; this version posted April 3, 2026. The copyright holder for this preprint (which was not certified by peer review) is the author/funder, who has granted bioRxiv a license to display the preprint in perpetuity. It is made available under aCC-BY 4.0 International license.

- 0.02% (v/v) sodium azide

All components were mixed thoroughly until fully dissolved. Buffer was stored at 4°C for up to 1 month.

Secondary Antibody Buffer

The secondary antibody buffer (NGSTU-azide) was prepared in 1×PBS with the following composition:

- 5% (v/v) normal goat serum
- 0.6% (v/v) Triton X-100
- 0.02% (v/v) sodium azide

All components were mixed thoroughly until fully dissolved. Buffer was stored at 4°C for up to 1 month.

Expanding Hydrogel Monomer Solution

The expanding hydrogel monomer stock solution was prepared using the following components *(adapted from Tavakoli et al., 2025):*

- 10% (w/v) acrylamide
- 12.5% (w/v) sodium acrylate
- 0.075% (w/v) N,N′-bisacrylamide

All components were dissolved in Milli-Q water. The solution was vortexed thoroughly and centrifuged at 4,500 × g for 5 minutes to remove particulate matter. The resulting supernatant was transferred to a fresh 50 mL conical tube, aliquoted into 1 mL volumes, and stored at −20 °C for up to one month.

Digestion Buffer

(adapted from Tillberg et al., 2016)

- 100 mM Tris base
- 5% (v/v) Triton X-100
- 1% (w/v) sodium dodecyl sulfate (SDS)

All components were dissolved in Milli-Q water, and the solution was mixed thoroughly until fully solubilized. The buffer was prepared fresh or stored at room temperature for short-term use. Prior to use, the buffer was equilibrated to 37 °C. This solution facilitates enzymatic digestion and mechanical disruption of the tissue-hydrogel composite to allow uniform and isotropic expansion.
